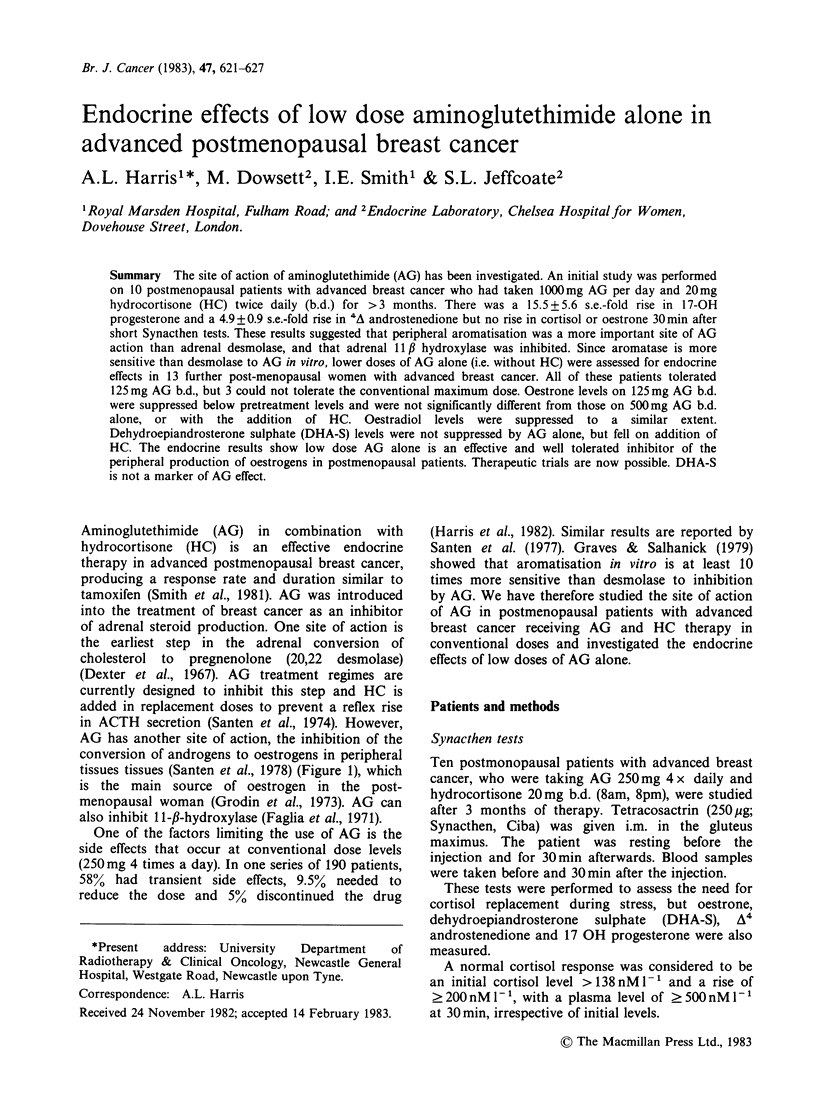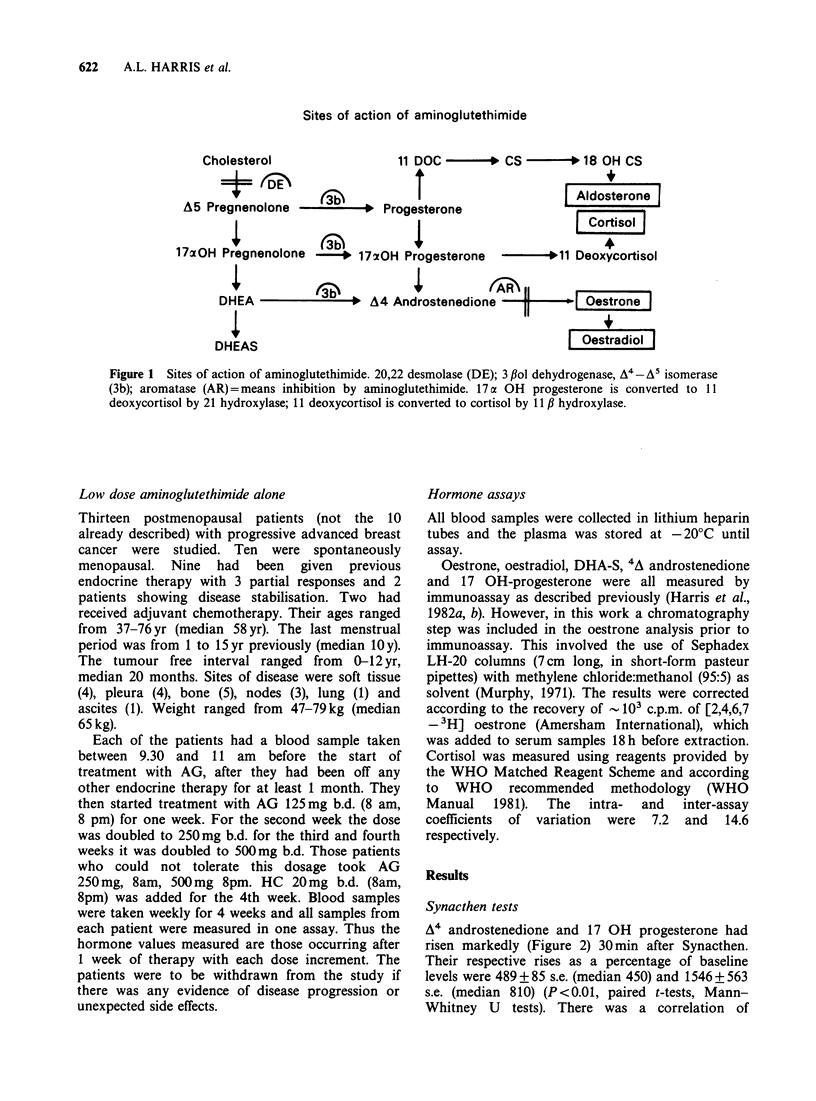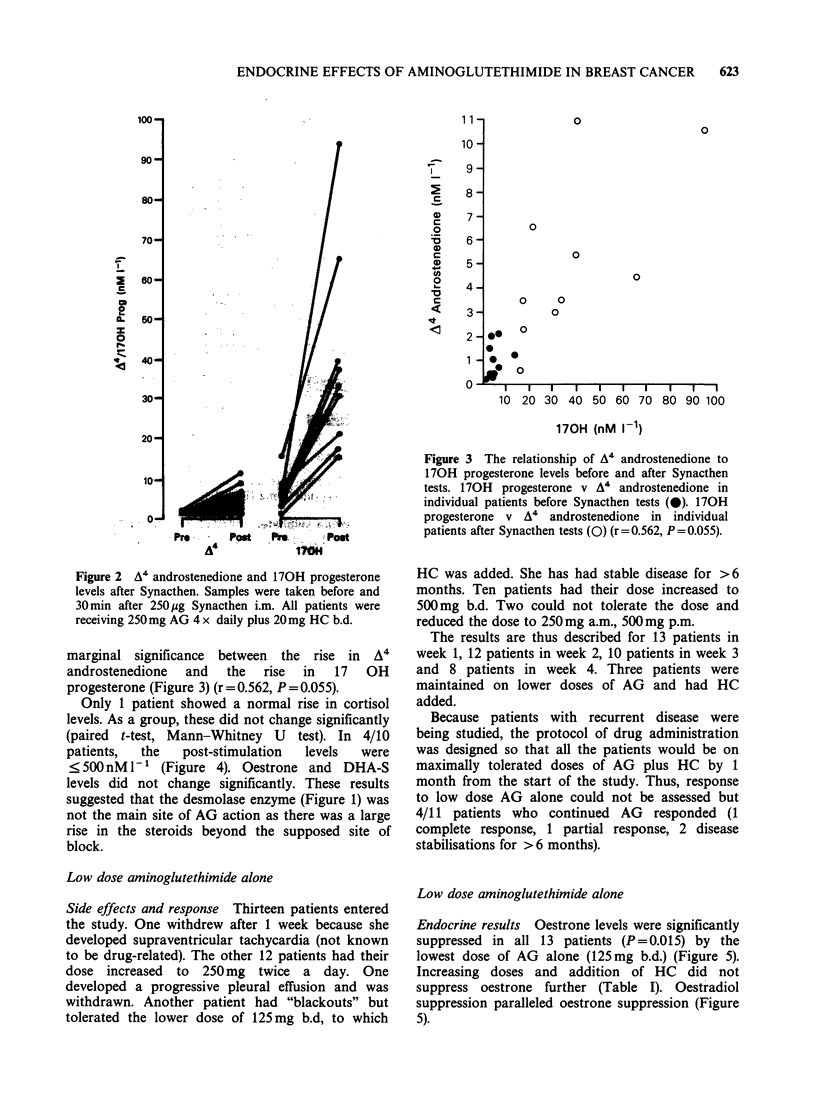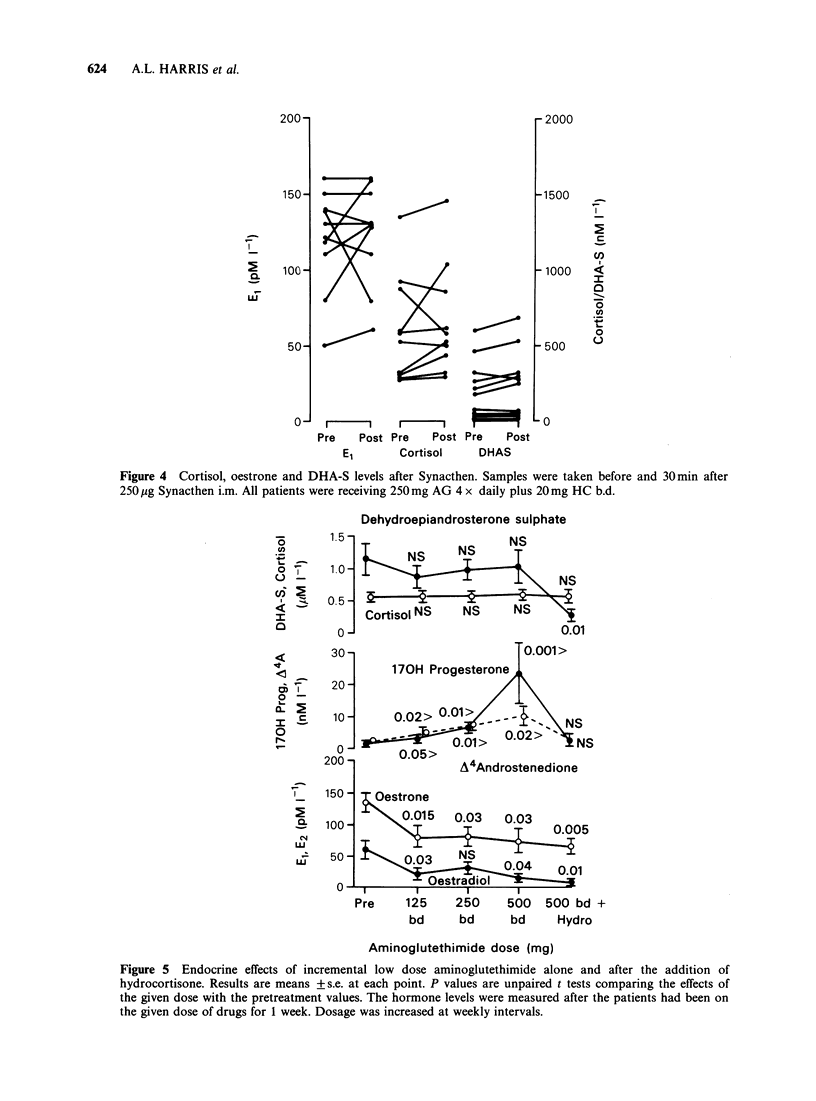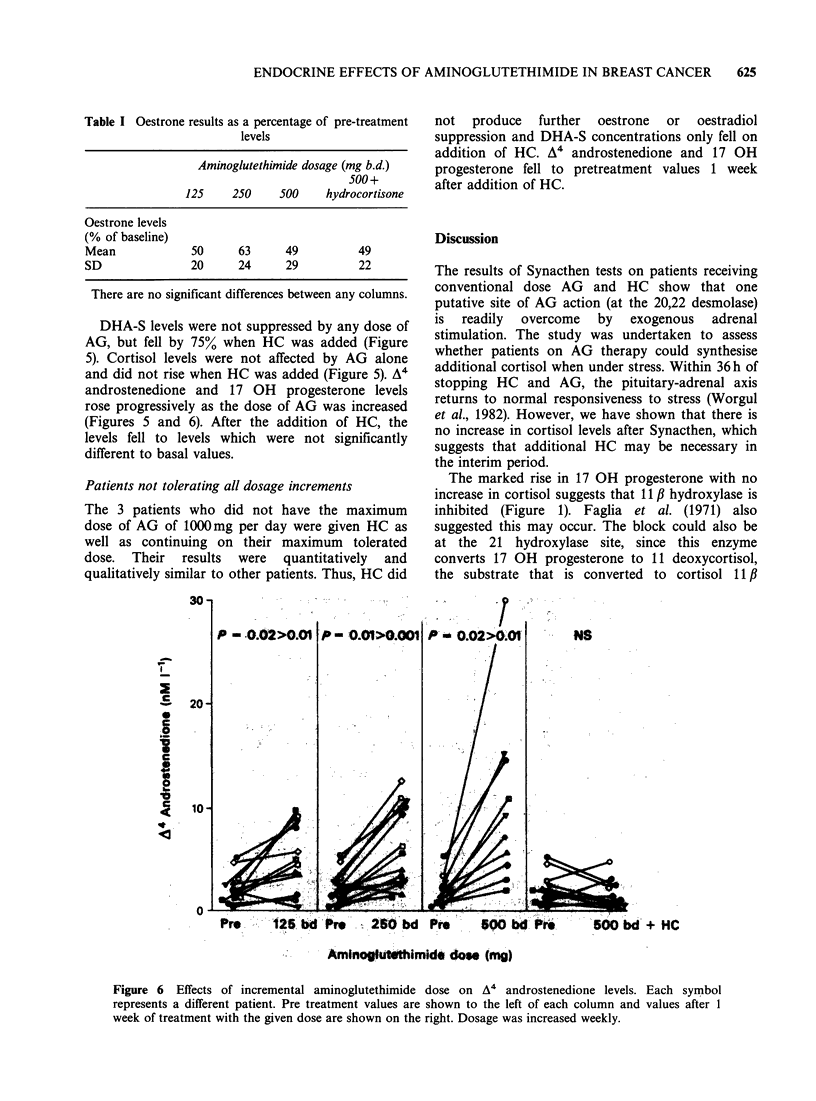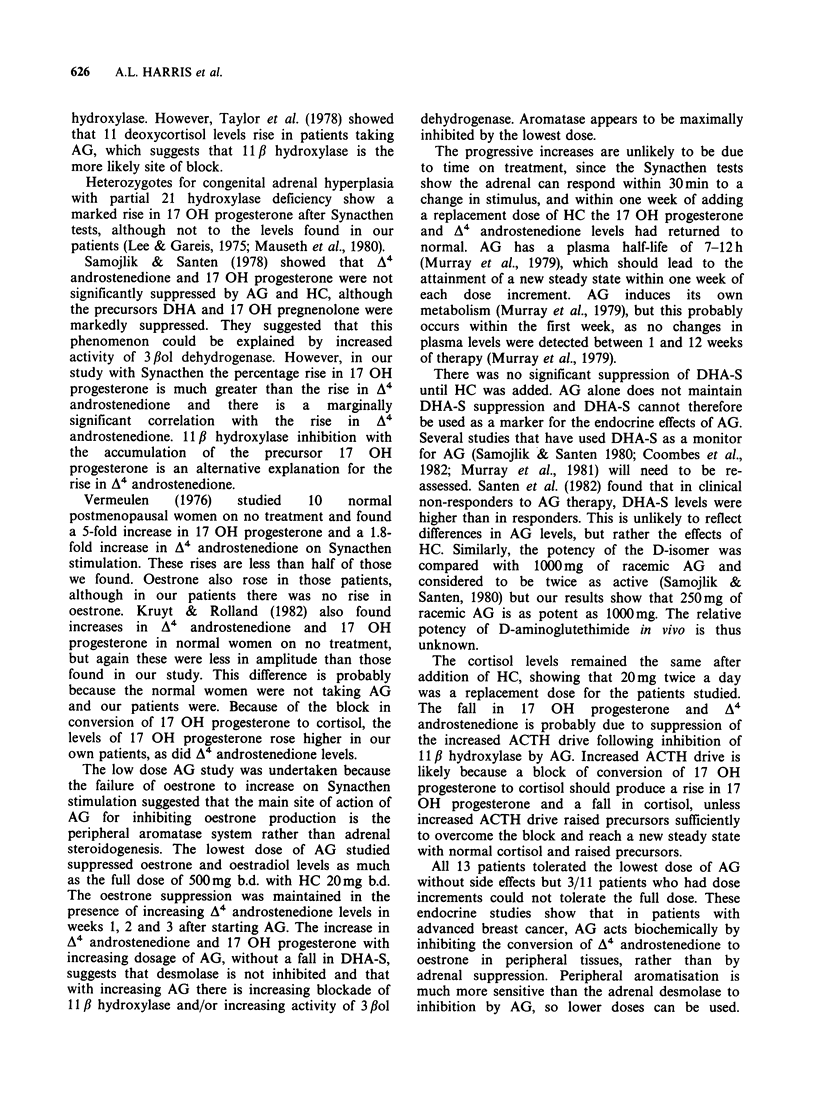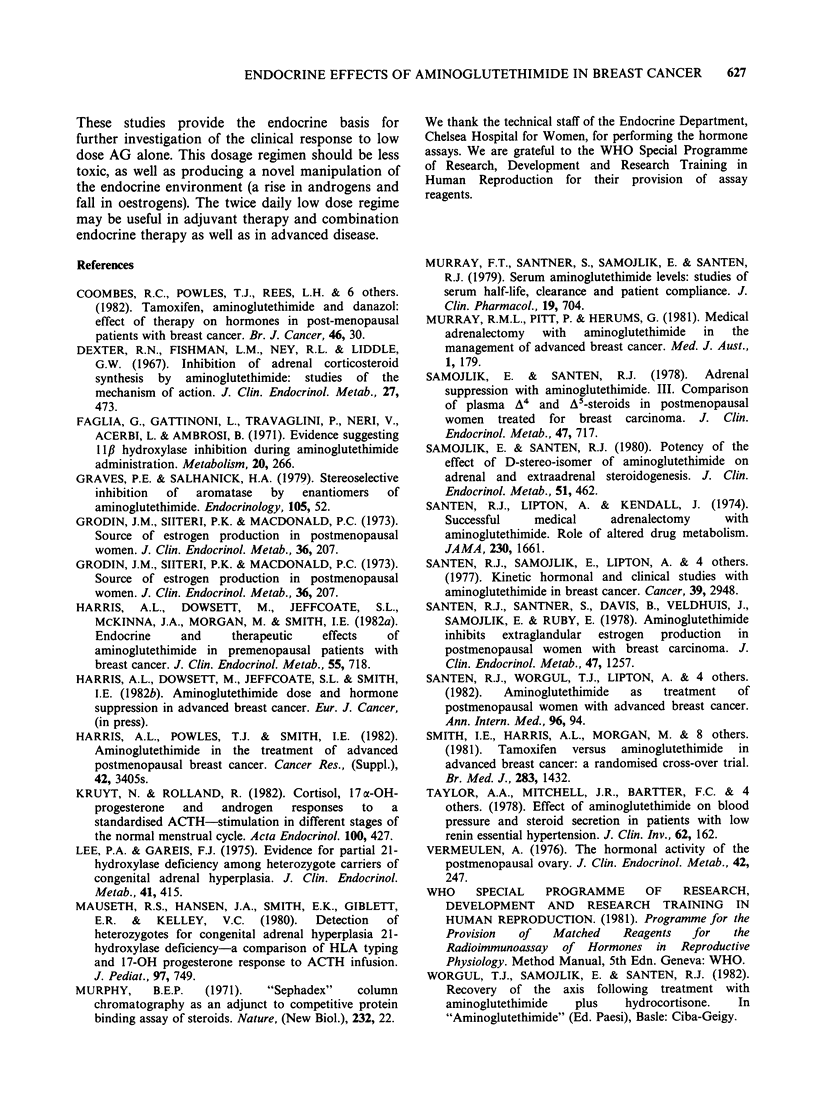# Endocrine effects of low dose aminoglutethimide alone in advanced postmenopausal breast cancer.

**DOI:** 10.1038/bjc.1983.100

**Published:** 1983-05

**Authors:** A. L. Harris, M. Dowsett, I. E. Smith, S. L. Jeffcoate

## Abstract

The site of action of aminoglutethimide (AG) has been investigated. An initial study was performed on 10 postmenopausal patients with advanced breast cancer who had taken 1000 mg AG per day and 20 mg hydrocortisone (HC) twice daily (b.d.) for greater than 3 months. There was a 15.5 +/- 5.6 s.e.-fold rise in 17-OH progesterone and a 4.9 +/- 0.9 s.e.-fold rise in 4 delta androstenedione but no rise in cortisol or oestrone 30 min after short Synacthen tests. These results suggested that peripheral aromatisation was a more important site of AG action than adrenal desmolase, and that adrenal 11 beta hydroxylase was inhibited. Since aromatase is more sensitive than desmolase to AG in vitro, lower doses of AG alone (i.e. without HC) were assessed for endocrine effects in 13 further post-menopausal women with advanced breast cancer. All of these patients tolerated 125 mg AG b.d., but 3 could not tolerate the conventional maximum dose. Oestrone levels on 125 mg AG b.d. were suppressed below pretreatment levels and were not significantly different from those on 500 mg AG b.d. alone, or with the addition of HC. Oestradiol levels were suppressed to a similar extent. Dehydroepiandrosterone sulphate (DHA-S) levels were not suppressed by AG alone, but fell on addition of HC. The endocrine results show low dose AG alone is an effective and well tolerated inhibitor of the peripheral production of oestrogens in postmenopausal patients. Therapeutic trials are now possible. DHA-S is not a marker of AG effect.


*Br. J. Cancer* (1983), **47**, 621–627

### Endocrine effects of low dose aminoglutethimide alone in advanced postmenopausal breast cancer

A.L. Harris[1]*, M. Dowsett[2], I.E. Smith[1] & S.L. Jeffcoate[2]

[1]*Royal Marsden Hospital, Fulham Road; and* [2]*Endocrine Laboratory, Chelsea Hospital for Women, Dovehouse Street, London.*

**Summary** The site of action of aminoglutethimide (AG) has been investigated. An initial study was performed on 10 postmenopausal patients with advanced breast cancer who had taken 1000 mg AG per day and 20 mg hydrocortisone (HC) twice daily (b.d.) for >3 months. There was a 15.5±5.6 s.e.-fold rise in 17-OH progesterone and a 4.9±0.9 s.e.-fold rise in $^4\Delta$ androstenedione but no rise in cortisol or oestrone 30 min after short Synacthen tests. These results suggested that peripheral aromatisation was a more important site of AG action than adrenal desmolase, and that adrenal 11$\beta$ hydroxylase was inhibited. Since aromatase is more sensitive than desmolase to AG *in vitro*, lower doses of AG alone (i.e. without HC) were assessed for endocrine effects in 13 further post-menopausal women with advanced breast cancer. All of these patients tolerated 125 mg AG b.d., but 3 could not tolerate the conventional maximum dose. Oestrone levels on 125 mg AG b.d. were suppressed below pretreatment levels and were not significantly different from those on 500 mg AG b.d. alone, or with the addition of HC. Oestradiol levels were suppressed to a similar extent. Dehydroepiandrosterone sulphate (DHA-S) levels were not suppressed by AG alone, but fell on addition of HC. The endocrine results show low dose AG alone is an effective and well tolerated inhibitor of the peripheral production of oestrogens in postmenopausal patients. Therapeutic trials are now possible. DHA-S is not a marker of AG effect.

Aminoglutethimide (AG) in combination with hydrocortisone (HC) is an effective endocrine therapy in advanced postmenopausal breast cancer, producing a response rate and duration similar to tamoxifen (Smith *et al.*, 1981). AG was introduced into the treatment of breast cancer as an inhibitor of adrenal steroid production. One site of action is the earliest step in the adrenal conversion of cholesterol to pregnenolone (20,22 desmolase) (Dexter *et al.*, 1967). AG treatment regimes are currently designed to inhibit this step and HC is added in replacement doses to prevent a reflex rise in ACTH secretion (Santen *et al.*, 1974). However, AG has another site of action, the inhibition of the conversion of androgens to oestrogens in peripheral tissues tissues (Santen *et al.*, 1978) (Figure 1), which is the main source of oestrogen in the post-menopausal woman (Grodin *et al.*, 1973). AG can also inhibit 11-$\beta$-hydroxylase (Faglia *et al.*, 1971).

One of the factors limiting the use of AG is the side effects that occur at conventional dose levels (250 mg 4 times a day). In one series of 190 patients, 58% had transient side effects, 9.5% needed to reduce the dose and 5% discontinued the drug (Harris *et al.*, 1982). Similar results are reported by Santen *et al.* (1977). Graves & Salhanick (1979) showed that aromatisation *in vitro* is at least 10 times more sensitive than desmolase to inhibition by AG. We have therefore studied the site of action of AG in postmenopausal patients with advanced breast cancer receiving AG and HC therapy in conventional doses and investigated the endocrine effects of low doses of AG alone.

*Present address: University Department of Radiotherapy & Clinical Oncology, Newcastle General Hospital, Westgate Road, Newcastle upon Tyne.

Correspondence: A.L. Harris

Received 24 November 1982; accepted 14 February 1983.

### Patients and methods

### *Synacthen tests*

Ten postmonopausal patients with advanced breast cancer, who were taking AG 250 mg 4× daily and hydrocortisone 20 mg b.d. (8am, 8pm), were studied after 3 months of therapy. Tetracosactrin (250 $\mu$g; Synacthen, Ciba) was given i.m. in the gluteus maximus. The patient was resting before the injection and for 30 min afterwards. Blood samples were taken before and 30 min after the injection.

These tests were performed to assess the need for cortisol replacement during stress, but oestrone, dehydroepiandrosterone sulphate (DHA-S), $\Delta^4$ androstenedione and 17 OH progesterone were also measured.

A normal cortisol response was considered to be an initial cortisol level >138 nM l$^{-1}$ and a rise of ≥200 nM l$^{-1}$, with a plasma level of ≥500 nM l$^{-1}$ at 30 min, irrespective of initial levels.

© The Macmillan Press Ltd., 1983

**Figure 1** Sites of action of aminoglutethimide. 20,22 desmolase (DE); 3βol dehydrogenase, $\Delta^4-\Delta^5$ isomerase (3b); aromatase (AR) = means inhibition by aminoglutethimide. 17α OH progesterone is converted to 11 deoxycortisol by 21 hydroxylase; 11 deoxycortisol is converted to cortisol by 11β hydroxylase.

*Low dose aminoglutethimide alone*

Thirteen postmenopausal patients (not the 10 already described) with progressive advanced breast cancer were studied. Ten were spontaneously menopausal. Nine had been given previous endocrine therapy with 3 partial responses and 2 patients showing disease stabilisation. Two had received adjuvant chemotherapy. Their ages ranged from 37–76 yr (median 58 yr). The last menstrual period was from 1 to 15 yr previously (median 10 y). The tumour free interval ranged from 0–12 yr, median 20 months. Sites of disease were soft tissue (4), pleura (4), bone (5), nodes (3), lung (1) and ascites (1). Weight ranged from 47–79 kg (median 65 kg).

Each of the patients had a blood sample taken between 9.30 and 11 am before the start of treatment with AG, after they had been off any other endocrine therapy for at least 1 month. They then started treatment with AG 125 mg b.d. (8 am, 8 pm) for one week. For the second week the dose was doubled to 250 mg b.d. for the third and fourth weeks it was doubled to 500 mg b.d. Those patients who could not tolerate this dosage took AG 250 mg, 8am, 500 mg 8pm. HC 20 mg b.d. (8am, 8pm) was added for the 4th week. Blood samples were taken weekly for 4 weeks and all samples from each patient were measured in one assay. Thus the hormone values measured are those occurring after 1 week of therapy with each dose increment. The patients were to be withdrawn from the study if there was any evidence of disease progression or unexpected side effects.

*Hormone assays*

All blood samples were collected in lithium heparin tubes and the plasma was stored at −20°C until assay.

Oestrone, oestradiol, DHA-S, $^4\Delta$ androstenedione and 17 OH-progesterone were all measured by immunoassay as described previously (Harris *et al.*, 1982*a*, *b*). However, in this work a chromatography step was included in the oestrone analysis prior to immunoassay. This involved the use of Sephadex LH-20 columns (7 cm long, in short-form pasteur pipettes) with methylene chloride:methanol (95:5) as solvent (Murphy, 1971). The results were corrected according to the recovery of $\sim 10^3$ c.p.m. of [2,4,6,7 $-{}^3$H] oestrone (Amersham International), which was added to serum samples 18 h before extraction. Cortisol was measured using reagents provided by the WHO Matched Reagent Scheme and according to WHO recommended methodology (WHO Manual 1981). The intra- and inter-assay coefficients of variation were 7.2 and 14.6 respectively.

### Results

*Synacthen tests*

$\Delta^4$ androstenedione and 17 OH progesterone had risen markedly (Figure 2) 30 min after Synacthen. Their respective rises as a percentage of baseline levels were $489 \pm 85$ s.e. (median 450) and $1546 \pm 563$ s.e. (median 810) ($P < 0.01$, paired *t*-tests, Mann–Whitney U tests). There was a correlation of

**Figure 2** $\Delta^4$ androstenedione and 17OH progesterone levels after Synacthen. Samples were taken before and 30 min after 250 $\mu$g Synacthen i.m. All patients were receiving 250 mg AG 4 × daily plus 20 mg HC b.d.

**Figure 3** The relationship of $\Delta^4$ androstenedione to 17OH progesterone levels before and after Synacthen tests. 17OH progesterone v $\Delta^4$ androstenedione in individual patients before Synacthen tests (●). 17OH progesterone v $\Delta^4$ androstenedione in individual patients after Synacthen tests (○) ($r = 0.562$, $P = 0.055$).

marginal significance between the rise in $\Delta^4$ androstenedione and the rise in 17 OH progesterone (Figure 3) ($r = 0.562$, $P = 0.055$).

Only 1 patient showed a normal rise in cortisol levels. As a group, these did not change significantly (paired *t*-test, Mann–Whitney U test). In 4/10 patients, the post-stimulation levels were $\leq 500\,\mathrm{nM\,l^{-1}}$ (Figure 4). Oestrone and DHA-S levels did not change significantly. These results suggested that the desmolase enzyme (Figure 1) was not the main site of AG action as there was a large rise in the steroids beyond the supposed site of block.

*Low dose aminoglutethimide alone*

*Side effects and response* Thirteen patients entered the study. One withdrew after 1 week because she developed supraventricular tachycardia (not known to be drug-related). The other 12 patients had their dose increased to 250 mg twice a day. One developed a progressive pleural effusion and was withdrawn. Another patient had "blackouts" but tolerated the lower dose of 125 mg b.d, to which HC was added. She has had stable disease for >6 months. Ten patients had their dose increased to 500 mg b.d. Two could not tolerate the dose and reduced the dose to 250 mg a.m., 500 mg p.m.

The results are thus described for 13 patients in week 1, 12 patients in week 2, 10 patients in week 3 and 8 patients in week 4. Three patients were maintained on lower doses of AG and had HC added.

Because patients with recurrent disease were being studied, the protocol of drug administration was designed so that all the patients would be on maximally tolerated doses of AG plus HC by 1 month from the start of the study. Thus, response to low dose AG alone could not be assessed but 4/11 patients who continued AG responded (1 complete response, 1 partial response, 2 disease stabilisations for >6 months).

*Low dose aminoglutethimide alone*

*Endocrine results* Oestrone levels were significantly suppressed in all 13 patients ($P = 0.015$) by the lowest dose of AG alone (125 mg b.d.) (Figure 5). Increasing doses and addition of HC did not suppress oestrone further (Table I). Oestradiol suppression paralleled oestrone suppression (Figure 5).

**Figure 4** Cortisol, oestrone and DHA-S levels after Synacthen. Samples were taken before and 30 min after 250 μg Synacthen i.m. All patients were receiving 250 mg AG 4 × daily plus 20 mg HC b.d.

**Figure 5** Endocrine effects of incremental low dose aminoglutethimide alone and after the addition of hydrocortisone. Results are means ±s.e. at each point. *P* values are unpaired *t* tests comparing the effects of the given dose with the pretreatment values. The hormone levels were measured after the patients had been on the given dose of drugs for 1 week. Dosage was increased at weekly intervals.

**Table I** Oestrone results as a percentage of pre-treatment levels

| | *Aminoglutethimide dosage (mg b.d.)* | | | |
|---|---|---|---|---|
| | *125* | *250* | *500* | *500+ hydrocortisone* |
| Oestrone levels (% of baseline) | | | | |
| Mean | 50 | 63 | 49 | 49 |
| SD | 20 | 24 | 29 | 22 |

There are no significant differences between any columns.

DHA-S levels were not suppressed by any dose of AG, but fell by 75% when HC was added (Figure 5). Cortisol levels were not affected by AG alone and did not rise when HC was added (Figure 5). $\Delta^4$ androstenedione and 17 OH progesterone levels rose progressively as the dose of AG was increased (Figures 5 and 6). After the addition of HC, the levels fell to levels which were not significantly different to basal values.

*Patients not tolerating all dosage increments*

The 3 patients who did not have the maximum dose of AG of 1000 mg per day were given HC as well as continuing on their maximum tolerated dose. Their results were quantitatively and qualitatively similar to other patients. Thus, HC did not produce further oestrone or oestradiol suppression and DHA-S concentrations only fell on addition of HC. $\Delta^4$ androstenedione and 17 OH progesterone fell to pretreatment values 1 week after addition of HC.

### Discussion

The results of Synacthen tests on patients receiving conventional dose AG and HC show that one putative site of AG action (at the 20,22 desmolase) is readily overcome by exogenous adrenal stimulation. The study was undertaken to assess whether patients on AG therapy could synthesise additional cortisol when under stress. Within 36 h of stopping HC and AG, the pituitary-adrenal axis returns to normal responsiveness to stress (Worgul *et al.*, 1982). However, we have shown that there is no increase in cortisol levels after Synacthen, which suggests that additional HC may be necessary in the interim period.

The marked rise in 17 OH progesterone with no increase in cortisol suggests that 11 $\beta$ hydroxylase is inhibited (Figure 1). Faglia *et al.* (1971) also suggested this may occur. The block could also be at the 21 hydroxylase site, since this enzyme converts 17 OH progesterone to 11 deoxycortisol, the substrate that is converted to cortisol 11 $\beta$

**Figure 6** Effects of incremental aminoglutethimide dose on $\Delta^4$ androstenedione levels. Each symbol represents a different patient. Pre treatment values are shown to the left of each column and values after 1 week of treatment with the given dose are shown on the right. Dosage was increased weekly.

hydroxylase. However, Taylor *et al.* (1978) showed that 11 deoxycortisol levels rise in patients taking AG, which suggests that $11\beta$ hydroxylase is the more likely site of block.

Heterozygotes for congenital adrenal hyperplasia with partial 21 hydroxylase deficiency show a marked rise in 17 OH progesterone after Synacthen tests, although not to the levels found in our patients (Lee & Gareis, 1975; Mauseth *et al.*, 1980).

Samojlik & Santen (1978) showed that $\Delta^4$ androstenedione and 17 OH progesterone were not significantly suppressed by AG and HC, although the precursors DHA and 17 OH pregnenolone were markedly suppressed. They suggested that this phenomenon could be explained by increased activity of $3\beta$ol dehydrogenase. However, in our study with Synacthen the percentage rise in 17 OH progesterone is much greater than the rise in $\Delta^4$ androstenedione and there is a marginally significant correlation with the rise in $\Delta^4$ androstenedione. $11\beta$ hydroxylase inhibition with the accumulation of the precursor 17 OH progesterone is an alternative explanation for the rise in $\Delta^4$ androstenedione.

Vermeulen (1976) studied 10 normal postmenopausal women on no treatment and found a 5-fold increase in 17 OH progesterone and a 1.8-fold increase in $\Delta^4$ androstenedione on Synacthen stimulation. These rises are less than half of those we found. Oestrone also rose in those patients, although in our patients there was no rise in oestrone. Kruyt & Rolland (1982) also found increases in $\Delta^4$ androstenedione and 17 OH progesterone in normal women on no treatment, but again these were less in amplitude than those found in our study. This difference is probably because the normal women were not taking AG and our patients were. Because of the block in conversion of 17 OH progesterone to cortisol, the levels of 17 OH progesterone rose higher in our own patients, as did $\Delta^4$ androstenedione levels.

The low dose AG study was undertaken because the failure of oestrone to increase on Synacthen stimulation suggested that the main site of action of AG for inhibiting oestrone production is the peripheral aromatase system rather than adrenal steroidogenesis. The lowest dose of AG studied suppressed oestrone and oestradiol levels as much as the full dose of 500 mg b.d. with HC 20 mg b.d. The oestrone suppression was maintained in the presence of increasing $\Delta^4$ androstenedione levels in weeks 1, 2 and 3 after starting AG. The increase in $\Delta^4$ androstenedione and 17 OH progesterone with increasing dosage of AG, without a fall in DHA-S, suggests that desmolase is not inhibited and that with increasing AG there is increasing blockade of $11\beta$ hydroxylase and/or increasing activity of $3\beta$ol dehydrogenase. Aromatase appears to be maximally inhibited by the lowest dose.

The progressive increases are unlikely to be due to time on treatment, since the Synacthen tests show the adrenal can respond within 30 min to a change in stimulus, and within one week of adding a replacement dose of HC the 17 OH progesterone and $\Delta^4$ androstenedione levels had returned to normal. AG has a plasma half-life of 7–12 h (Murray *et al.*, 1979), which should lead to the attainment of a new steady state within one week of each dose increment. AG induces its own metabolism (Murray *et al.*, 1979), but this probably occurs within the first week, as no changes in plasma levels were detected between 1 and 12 weeks of therapy (Murray *et al.*, 1979).

There was no significant suppression of DHA-S until HC was added. AG alone does not maintain DHA-S suppression and DHA-S cannot therefore be used as a marker for the endocrine effects of AG. Several studies that have used DHA-S as a monitor for AG (Samojlik & Santen 1980; Coombes *et al.*, 1982; Murray *et al.*, 1981) will need to be re-assessed. Santen *et al.* (1982) found that in clinical non-responders to AG therapy, DHA-S levels were higher than in responders. This is unlikely to reflect differences in AG levels, but rather the effects of HC. Similarly, the potency of the D-isomer was compared with 1000 mg of racemic AG and considered to be twice as active (Samojlik & Santen, 1980) but our results show that 250 mg of racemic AG is as potent as 1000 mg. The relative potency of D-aminoglutethimide *in vivo* is thus unknown.

The cortisol levels remained the same after addition of HC, showing that 20 mg twice a day was a replacement dose for the patients studied. The fall in 17 OH progesterone and $\Delta^4$ androstenedione is probably due to suppression of the increased ACTH drive following inhibition of $11\beta$ hydroxylase by AG. Increased ACTH drive is likely because a block of conversion of 17 OH progesterone to cortisol should produce a rise in 17 OH progesterone and a fall in cortisol, unless increased ACTH drive raised precursors sufficiently to overcome the block and reach a new steady state with normal cortisol and raised precursors.

All 13 patients tolerated the lowest dose of AG without side effects but 3/11 patients who had dose increments could not tolerate the full dose. These endocrine studies show that in patients with advanced breast cancer, AG acts biochemically by inhibiting the conversion of $\Delta^4$ androstenedione to oestrone in peripheral tissues, rather than by adrenal suppression. Peripheral aromatisation is much more sensitive than the adrenal desmolase to inhibition by AG, so lower doses can be used.

These studies provide the endocrine basis for further investigation of the clinical response to low dose AG alone. This dosage regimen should be less toxic, as well as producing a novel manipulation of the endocrine environment (a rise in androgens and fall in oestrogens). The twice daily low dose regime may be useful in adjuvant therapy and combination endocrine therapy as well as in advanced disease.

We thank the technical staff of the Endocrine Department, Chelsea Hospital for Women, for performing the hormone assays. We are grateful to the WHO Special Programme of Research, Development and Research Training in Human Reproduction for their provision of assay reagents.

### References

COOMBES, R.C., POWLES, T.J., REES, L.H. & 6 others. (1982). Tamoxifen, aminoglutethimide and danazol: effect of therapy on hormones in post-menopausal patients with breast cancer. *Br. J. Cancer*, **46**, 30.

DEXTER, R.N., FISHMAN, L.M., NEY, R.L. & LIDDLE, G.W. (1967). Inhibition of adrenal corticosteroid synthesis by aminoglutethimide: studies of the mechanism of action. *J. Clin. Endocrinol. Metab.*, **27**, 473.

FAGLIA, G., GATTINONI, L., TRAVAGLINI, P., NERI, V., ACERBI, L. & AMBROSI, B. (1971). Evidence suggesting 11$\beta$ hydroxylase inhibition during aminoglutethimide administration. *Metabolism*, **20**, 266.

GRAVES, P.E. & SALHANICK, H.A. (1979). Stereoselective inhibition of aromatase by enantiomers of aminoglutethimide. *Endocrinology*, **105**, 52.

GRODIN, J.M., SIITERI, P.K. & MACDONALD, P.C. (1973). Source of estrogen production in postmenopausal women. *J. Clin. Endocrinol. Metab.*, **36**, 207.

GRODIN, J.M., SIITERI, P.K. & MACDONALD, P.C. (1973). Source of estrogen production in postmenopausal women. *J. Clin. Endocrinol. Metab.*, **36**, 207.

HARRIS, A.L., DOWSETT, M., JEFFCOATE, S.L., MCKINNA, J.A., MORGAN, M. & SMITH, I.E. (1982*a*). Endocrine and therapeutic effects of aminoglutethimide in premenopausal patients with breast cancer. *J. Clin. Endocrinol. Metab.*, **55**, 718.

HARRIS, A.L., DOWSETT, M., JEFFCOATE, S.L. & SMITH, I.E. (1982*b*). Aminoglutethimide dose and hormone suppression in advanced breast cancer. *Eur. J. Cancer*, (in press).

HARRIS, A.L., POWLES, T.J. & SMITH, I.E. (1982). Aminoglutethimide in the treatment of advanced postmenopausal breast cancer. *Cancer Res.*, (Suppl.), **42**, 3405s.

KRUYT, N. & ROLLAND, R. (1982). Cortisol, 17$\alpha$-OH-progesterone and androgen responses to a standardised ACTH—stimulation in different stages of the normal menstrual cycle. *Acta Endocrinol.* **100**, 427.

LEE, P.A. & GAREIS, F.J. (1975). Evidence for partial 21-hydroxylase deficiency among heterozygote carriers of congenital adrenal hyperplasia. *J. Clin. Endocrinol. Metab.*, **41**, 415.

MAUSETH, R.S., HANSEN, J.A., SMITH, E.K., GIBLETT, E.R. & KELLEY, V.C. (1980). Detection of heterozygotes for congenital adrenal hyperplasia 21-hydroxylase deficiency—a comparison of HLA typing and 17-OH progesterone response to ACTH infusion. *J. Pediat.*, **97**, 749.

MURPHY, B.E.P. (1971). "Sephadex" column chromatography as an adjunct to competitive protein binding assay of steroids. *Nature*, (New Biol.), **232**, 22.

MURRAY, F.T., SANTNER, S., SAMOJLIK, E. & SANTEN, R.J. (1979). Serum aminoglutethimide levels: studies of serum half-life, clearance and patient compliance. *J. Clin. Pharmacol.*, **19**, 704.

MURRAY, R.M.L., PITT, P. & HERUMS, G. (1981). Medical adrenalectomy with aminoglutethimide in the management of advanced breast cancer. *Med. J. Aust.*, **1**, 179.

SAMOJLIK, E. & SANTEN, R.J. (1978). Adrenal suppression with aminoglutethimide. III. Comparison of plasma $\Delta^4$ and $\Delta^5$-steroids in postmenopausal women treated for breast carcinoma. *J. Clin. Endocrinol. Metab.*, **47**, 717.

SAMOJLIK, E. & SANTEN, R.J. (1980). Potency of the effect of D-stereo-isomer of aminoglutethimide on adrenal and extraadrenal steroidogenesis. *J. Clin. Endocrinol. Metab.*, **51**, 462.

SANTEN, R.J., LIPTON, A. & KENDALL, J. (1974). Successful medical adrenalectomy with aminoglutethimide. Role of altered drug metabolism. *JAMA*, **230**, 1661.

SANTEN, R.J., SAMOJLIK, E., LIPTON, A. & 4 others. (1977). Kinetic hormonal and clinical studies with aminoglutethimide in breast cancer. *Cancer*, **39**, 2948.

SANTEN, R.J., SANTNER, S., DAVIS, B., VELDHUIS, J., SAMOJLIK, E. & RUBY, E. (1978). Aminoglutethimide inhibits extraglandular estrogen production in postmenopausal women with breast carcinoma. *J. Clin. Endocrinol. Metab.*, **47**, 1257.

SANTEN, R.J., WORGUL, T.J., LIPTON, A. & 4 others. (1982). Aminoglutethimide as treatment of postmenopausal women with advanced breast cancer. *Ann. Intern. Med.*, **96**, 94.

SMITH, I.E., HARRIS, A.L., MORGAN, M. & 8 others. (1981). Tamoxifen versus aminoglutethimide in advanced breast cancer: a randomised cross-over trial. *Br. Med. J.*, **283**, 1432.

TAYLOR, A.A., MITCHELL, J.R., BARTTER, F.C. & 4 others. (1978). Effect of aminoglutethimide on blood pressure and steroid secretion in patients with low renin essential hypertension. *J. Clin. Inv.*, **62**, 162.

VERMEULEN, A. (1976). The hormonal activity of the postmenopausal ovary. *J. Clin. Endocrinol. Metab.*, **42**, 247.

WHO SPECIAL PROGRAMME OF RESEARCH, DEVELOPMENT AND RESEARCH TRAINING IN HUMAN REPRODUCTION. (1981). *Programme for the Provision of Matched Reagents for the Radioimmunoassay of Hormones in Reproductive Physiology*. Method Manual, 5th Edn. Geneva: WHO.

WORGUL, T.J., SAMOJLIK, E. & SANTEN, R.J. (1982). Recovery of the axis following treatment with aminoglutethimide plus hydrocortisone. In "Aminoglutethimide" (Ed. Paesi), Basle: Ciba-Geigy.